# Classification of Tree Species in Overstorey Canopy of Subtropical Forest Using QuickBird Images

**DOI:** 10.1371/journal.pone.0125554

**Published:** 2015-05-15

**Authors:** Chinsu Lin, Sorin C. Popescu, Gavin Thomson, Khongor Tsogt, Chein-I Chang

**Affiliations:** 1 Department of Forestry and Natural Resources, National Chiayi University, 300 University Road, Chiayi 60004, Taiwan; 2 Department of Ecosystem Science and Management, Texas A&M University, College Station, TX, 77843, United States of America; 3 Department of Applied Foreign Languages, National Formosa University, 64, Wenhua Road, Huwei Township, Yunlin (63201), Taiwan; 4 Department of Computer Science and Electrical Engineering, University of Maryland, Baltimore County, Baltimore, MD, 21250, United States of America; University of Maryland at College Park, UNITED STATES

## Abstract

This paper proposes a supervised classification scheme to identify 40 tree species (2 coniferous, 38 broadleaf) belonging to 22 families and 36 genera in high spatial resolution QuickBird multispectral images (HMS). Overall kappa coefficient (OKC) and species conditional kappa coefficients (SCKC) were used to evaluate classification performance in training samples and estimate accuracy and uncertainty in test samples. Baseline classification performance using HMS images and vegetation index (VI) images were evaluated with an OKC value of 0.58 and 0.48 respectively, but performance improved significantly (up to 0.99) when used in combination with an HMS spectral-spatial texture image (SpecTex). One of the 40 species had very high conditional kappa coefficient performance (SCKC ≥ 0.95) using 4-band HMS and 5-band VIs images, but, only five species had lower performance (0.68 ≤ SCKC ≤ 0.94) using the SpecTex images. When SpecTex images were combined with a Visible Atmospherically Resistant Index (VARI), there was a significant improvement in performance in the training samples. The same level of improvement could not be replicated in the test samples indicating that a high degree of uncertainty exists in species classification accuracy which may be due to individual tree crown density, leaf greenness (inter-canopy gaps), and noise in the background environment (intra-canopy gaps). These factors increase uncertainty in the spectral texture features and therefore represent potential problems when using pixel-based classification techniques for multi-species classification.

## Introduction

The past few decades have witnessed an increase in research concerning classification of land cover/land use via high spatial resolution satellite multispectral imagery, such as SPOT5, IKONOS, QuickBird, WorldView-2 and GeoEye. High resolution images capture fine spatial details required for vegetation cover mapping. For example, the QuickBird and GeoEye satellites provide images with high spatial resolution pixel size in the range of 0.61m/2.44m and 0.41m/1.65m for panchromatic and multispectral (MS) data respectively. In addition, the spatial resolution of MS data can be upgraded to the decimeter level by a method of data fusion or pansharpening. Such high spatial resolution (HSR) images can be used to support forest management, for example, by examining biological habitat and estimating tree growth and harvestable timber volume. Other uses include delineation of tree crowns [1–5], tree parameter estimation [6, 7], biodiversity exploration [8], the identification of herbaceous plant species [9], and thinning scenario simulation in recreational forest management [10]. Recently, many studies have focused on species-level forest classification using various satellite images, such as airborne/satellite MS images [11–14], airborne lidar with MS images [15–18], and ground-based lidar or airborne/in situ hyperspectral data [19–24]. In addition to the above studies, research has also been conducted to assess species density and/or species richness [25], vegetation phenology [26, 27], forest succession [28], and invasive woody species [29, 30]. Interestingly, very little work has been done in the past using satellite images to identify and differentiate a large number of tree species, particularly, in tropical/subtropical forest where many tree species share the overstorey canopy. If overstorey species can be clearly identified, the forest carbon sink could be estimated more accurately according to tree species-based allometric formulas.

This paper explores the potential of using HSR QuickBird MS images (abbreviated as HMS) to identify multiple tree species. Two special features, i.e., spectral and texture information of the images were utilized based on two considerations. First, vegetation indices expand the limited spectral features of vegetation in the QuickBird HMS image and hold potential for tree classification. Second, leaf greenness, crown density (inter-canopy gaps), and background pixels (intra-canopy gaps) often cause noise and uncertainty in spectral analysis of satellite imagery. Therefore devising a method for pixel noise reduction may also increase the potential for tree classification. Four alternative classification schemes that combine input datasets and classifiers were examined in this study. The first used the original set of pansharpened 4-band image (HMS). The second applied a set of 5-band vegetation index images (HMS5VI) derived from the original HMS images. The third classification scheme used 4-band spectral-spatial textural images (SpecTex) obtained from HMS. The final scheme used 13-band image (HMS13B) that integrated HMS, HMS5VI, and SpecTec images. The classifiers applied to the classification schemes were maximum likelihood classifier, Mahalanobis distance, spectral angle mapper, support vector machine, spectral information divergence, and neural network. This paper aims to classify a large number of tree species (40) and has the following main objectives:

- Determine the baseline accuracy of the proposed classification scheme using training samples of only the original 4-band HMS QuickBird images and their derived vegetation indices.
- Examine if the baseline performance in the training samples can be improved by incorporating spatial texture features derived from the HMS images derived.
- Consider whether a marginal signature can be introduced to improve identification of hard to classify accurately (HCA) species in the training samples (i.e. the conditional kappa coefficient for any specific HCA species is less than 0.95).
- Evaluate the accuracy of classifications using the four datasets and classifiers by retraining with test samples.

## Materials and Methods

### Study site and species-based stratified sampling

**Fig 1:**
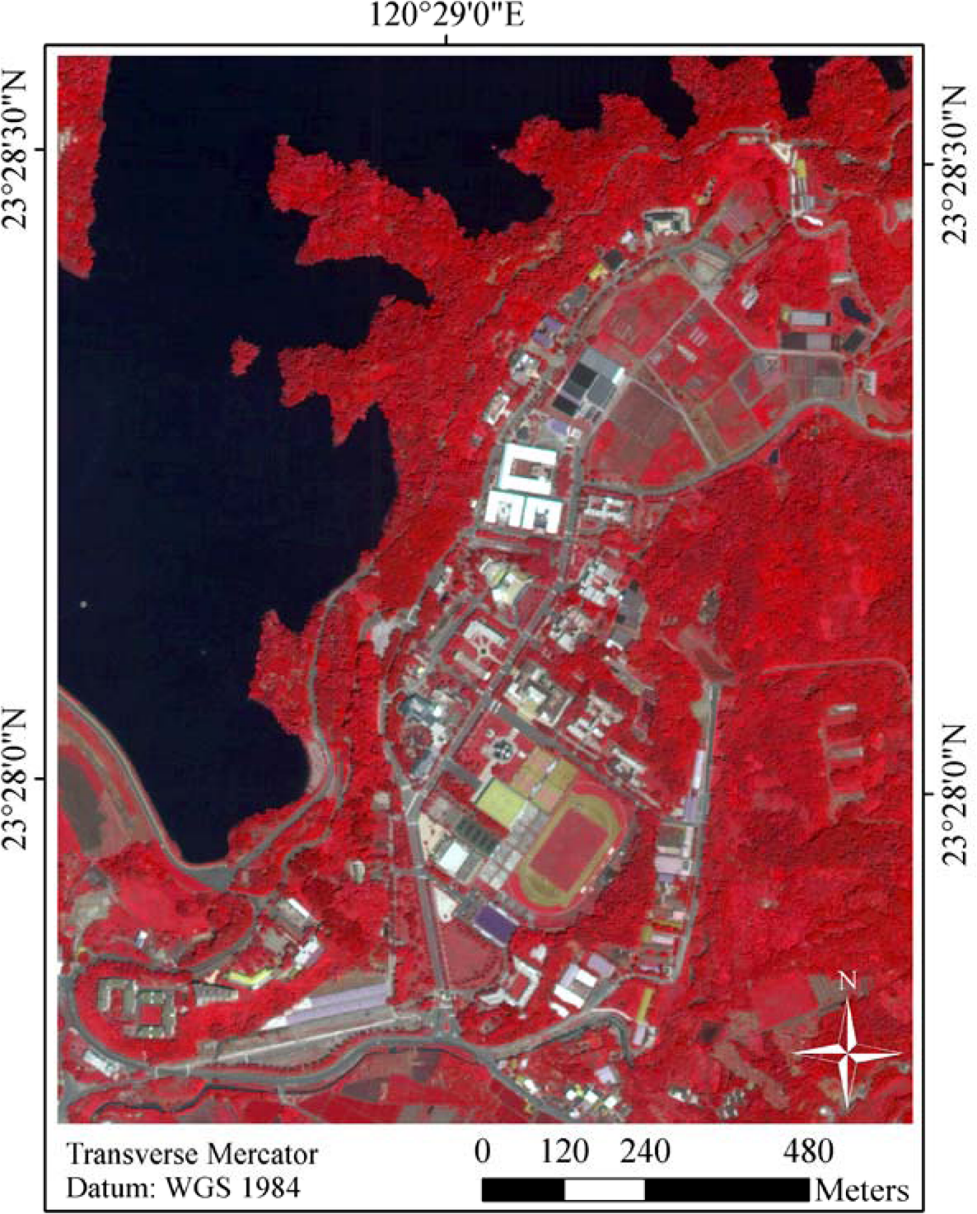
A false color picture of the QuickBird multispectral image (a composite of band 4, 3, 2 for red, green, and blue) showing the land covers over the study site.

**Fig 2:**
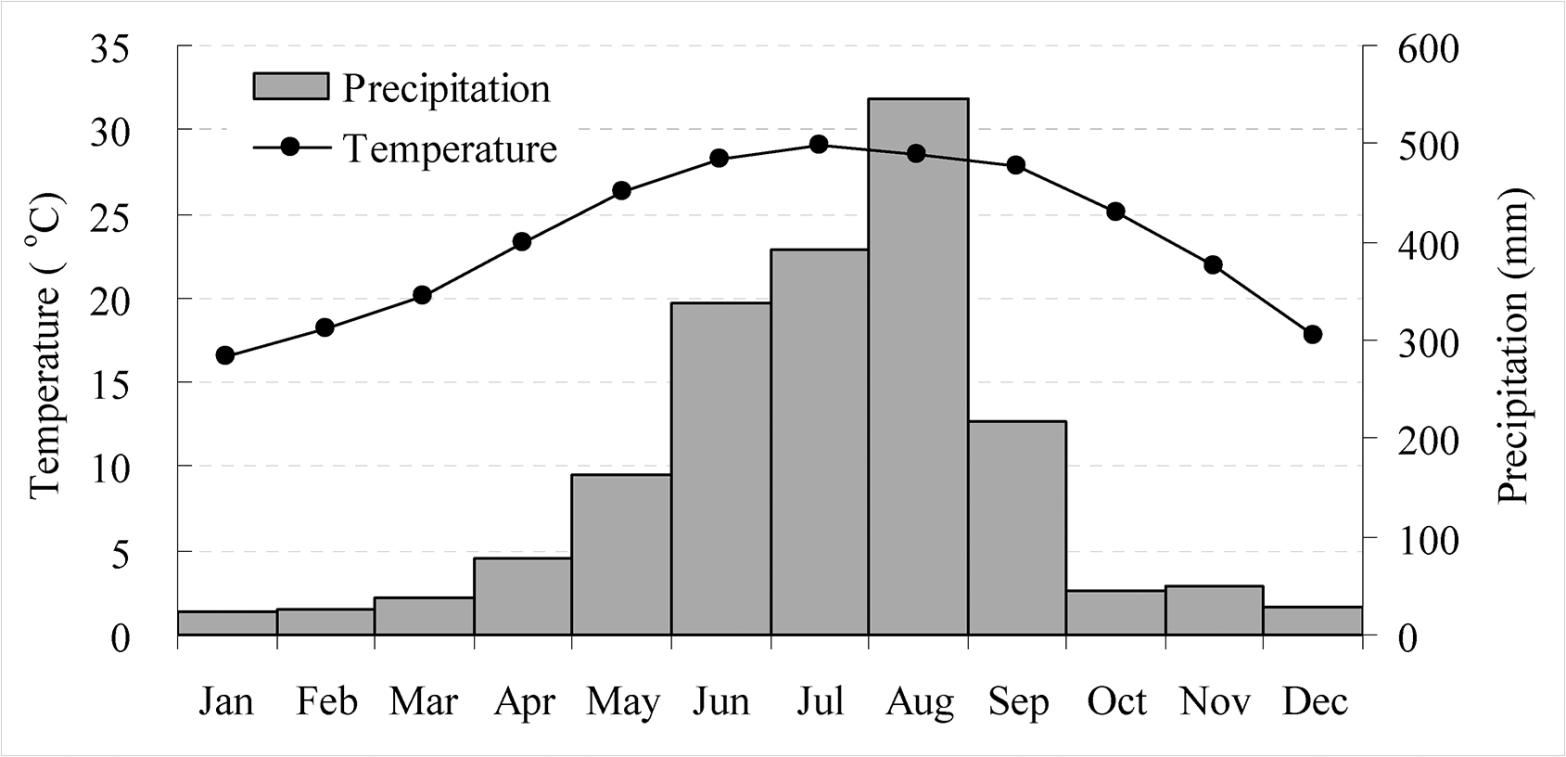
Generalized trends of the average monthly temperature (curve) and precipitation (bars) of the study site for the years 2006 to 2014.

Taiwan is located in a tropical/subtropical zone where 12 zonal and 9 azonal vegetation types are widely distributed. Diversity of forest vegetation in Taiwan is strongly influenced by temperature and moisture gradient [31]. The study site shown in Fig 1 is located in the watershed area of Lantan Dam in southern Taiwan centered at 120° 29’03”E and 23° 28’09”N. The average elevation is around 60m above sea level. Historic records from the weather station near the study site showed that the average 10-year annual temperature and precipitation of the area between 2006 and 2014 was 23.6°C and 1940mm respectively. As shown in Fig 2, average monthly temperature and precipitation varied from 17°C to 29°C and 23mm to 545mm and displayed a bell-shaped curve that peaked in July and August respectively.

A ground survey revealed that 40 tree species (Table 1) inhabited the forest overstorey canopy in the study site. A species-based stratified sampling method was applied to extract the identified tree species pixels as reference samples (N1 = 5727) for signature training. After training, an additional dataset also comprised of 40 species was randomly selected for use as test samples (N2 = 4102). Training and test samples were collected using a laser range finder and Leica GS5+ GPS which offers positioning accuracy up to one meter. The image resolution was sufficiently fine to identify the sample trees via image interpretation. In addition to explicitly identifying the neighborhood of the trees in the study site, each of the training and test samples was identified by double-checking the image interpretation with the on-site inventory. Table 1 tabulates the number of pixels of each species found in the training dataset and test dataset.

**Table 1:** Tree species used for spectral-spatial texture classification.

Family	Scientific name	Denoted by	Training samples	Test samples	Family	Scientific name	Denoted by	Training samples	Test samples
Mimosaceae	*Acacia auriculiformis*	*A*.*a*	60	67	Sapindaceae	*Koelreuteria formosana*	*K*.*f*	47	405
Palmae	*Areca catechu*	*A*.*ca*	77	330	Hamamelidaceae	*Liquidambar formosana*	*L*.*f*	89	40
Araucariaceae	*Araucaria cunninghamii*	*A*.*cu*	59	142	Mimosaceae	*Leucaena leucocephala*	*L*.*l*	197	60
Araucariaceae	*Araucaria excelsa*	*A*.*e*	40	159	Lythraceae	*Lagerstroemia subcostata*	*L*.*s*	112	58
Moraceae	*Artocarpus heterophyllus*	*A*.*h*	73	31	Myrtaceae	*Melaleuca leucadendea*	*M*.*l*	39	15
Apocynaceae	*Alstonia scholaris*	*A*.*s*	54	31	Euphorbiaceae	*Mallotus paniculatus*	*M*.*p*	75	25
Poaceae	*Bambuseae*	*B*.	633	81	Sapotaceae	*Palaquium formosanum*	*P*.*f*	46	55
Euphorbiaceae	*Bischofia iavanica*	*B*.*i*	40	20	Apocynaceae	*Plumeria rubra*	*P*.*r*	46	180
Caesalpiniaceae	*Cassia fistula*	*C*.*f*	70	69	Strelitziaceae	*Ravenala madagascariensis*	*R*.*m*	63	142
Fagales	*Cyclobalanopsis glauca*	*C*.*g*	55	349	Palmae	*Roystonea regia*	*R*.*r*	38	34
Apocynaceae	*Cerbera manghas*	*C*.*m*	32	26	Bignoniaceae	*Spathodea campanulata*	*S*.*c*	189	758
Annonaceae	*Cananga odorata*	*C*.*o*	315	41	Myrtaceae	*Syzygium formosanum*	*S*.*f*	108	27
Caesalpiniaceae	*Cassia siamea*	*C*.*si*	89	52	Sapindaceae	*Sapindus longana*	*S*.*l*	183	52
Caesalpiniaceae	*Cassia suratten*	*C*.*su*	196	37	Meliaceae	*Swietenia macrophylla*	*S*.*m*	150	64
Caesalpiniaceae	*Delonix regia*	*D*.*r*	274	69	Combretaceae	*Terminalia catappa*	*T*.*ca*	371	134
Fabaceae	*Dalbergia sissoo*	*D*.*s*	118	94	Bignoniaceae	*Tabebuia chrysantha*	*T*.*ch*	58	56
Moraceae	*Ficus eladtica*	*F*.*e*	296	45	Verbenaceae	*Tectona grandis*	*T*.*g*	45	25
Moraceae	*Ficus religiosa*	*F*.*r*	164	20	Ulmaceae	*Trema orientalis*	*T*.*o*	340	48
Caesalpiniaceae	*Haematoxylon campechianum*	*H*.*c*	262	45	Ulmaceae	*Ulmus parvifolia*	*U*.*p*	414	59
Palmae	*Heritiera littoralis*	*H*.*l*	36	16	Ulmaceae	*Zelkova serrata*	*Z*.*s*	122	141

### Procedures for multispectral image processing and analysis

**Fig 3:**
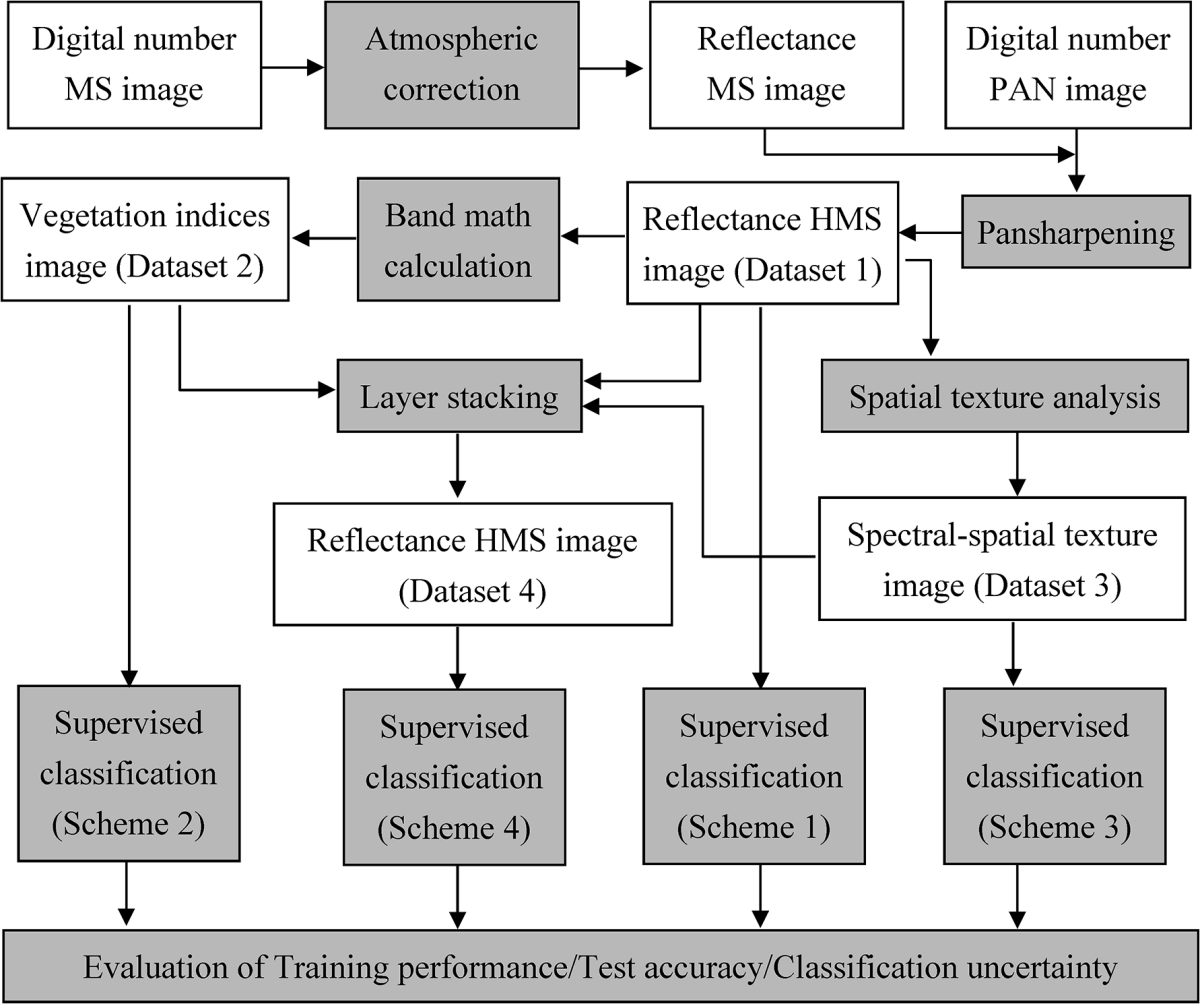
Flowchart of image processing and analyses used in this study.

QuickBird uses a pushbroom sensor to collect image data in a panchromatic band and 4 multispectral bands with a pixel size of 0.61m and 2.44m at nadir, respectively. The coverage of spectral bandwidths is blue: 450–520 nm, green: 520–600 nm, red: 630–690 nm, and near infrared: 760–900 nm, and panchromatic: 445–900 nm. The images used for this study were collected on 24 Aug, 2002, at 10:33am. The images were re-sampled to a pixel size of 0.6m and 2.4m for both the panchromatic and multispectral band images respectively after geometrical correction by the data distribution center. A series of image processing techniques such as atmospheric correction, data fusion (pansharpening), vegetation-index calculation, spatial-texture analysis, and layer stacking were carried out to produce variant datasets for species classification and the evaluation of training performance, test accuracy, and classification uncertainty (Fig 3). Please refer to the following sections for details of the image processing and analysis.

### Multispectral image preprocessing

#### Atmospheric correction

As shown in Eq (1), the digital number of the original 4-band multispectral image was first converted to at-sensor radiance *L*_*λ*,*i*_ (*Wm* ^−2^ *μm* ^−1^ *sr* ^−1^) using calibration coefficients (*a*_*i*_ and *k*_*i*_) and the nominal bandpass width of each band (Δ*λ*_*i*_) accompanying the metadata of the image (Table 2). Then *L*_*λ*,*i*_ was used to derive the reflectance (dimensionless) of surface materials by the atmospheric correction module (ATMOSC) in the commercial software IDRISI. The reflectance at the blue, green, red, and near infrared bands define the spectral signature of the material in a pixel.

$$L_{\lambda ,i}=\frac{L_{i}}{\Delta \lambda_{i}}=\frac{a_{i}\cdot k_{i}\cdot DN_{i}}{\Delta \lambda_{i}}$$ (1)

**Table 2:** Calibration coefficients for absolute radiance conversion of 16-bit QuickBird images^1)^.

Spectral band	Revised factor “k” (*Wm* ^−2^ *sr* ^−1^ *count* ^−1^)	Calibration gains “a” (*Wm* ^−2^ *sr* ^−1^ *count* ^−1^)	Nominal bandpass width (nm)
Pan	0.0838188	0.06269	455
Blue	0.0160412	0.01431	70
Green	0.0143847	0.01045	80
Red	0.0126735	0.00968	60
NIR	0.0154242	0.01568	140

^1)^ Revised factor for the panchromatic band varies with its exposure levels using time-delayed-integration (TDI); factor value is positively proportional to the TDI level, which can be found in. IMD files. Revised factor was set to 1 for each band for the product generated after June 6, 2003. Coefficients listed in the table address the case for 10 TDI levels. Absolute calibration coefficients for each band are also available from the. IMD file [32].

#### Data fusion

Geometrically registered QuickBird multispectral images (pixel size: 2.4m) were fused using principal components transformation (PCT)-based fusion techniques according to the following Eq (2) to create a QuickBird HMS image (pixel size: 0.6m).

$$H=\text{min}\;DN\_PC_{1}+\frac{\left(DN\_Pan-\text{min}\;DN\_Pan)(\text{max}\;DN\_PC_{1}-\text{min}\;DN\_PC_{1}\right)}{\left(\text{max}\;DN\_Pan-\text{min}\;DN\_Pan\right)}$$ (2)

The above PCT-based image fusion is a spectral domain technique with keeping the original spectral bands intact [33]. So, the fused HMS image obtained both the spectral and spatial information from the original QuickBird MS image and the panchromatic image for individual tree identification [34].

#### Expanding image spectral information by including vegetation indices

Recent efforts have employed several different vegetation indices (VIs) in modeling vegetation biophysical variables such as leaf area index (LAI), canopy closure or percentage green cover, green biomass, and chlorophyll content. Many VIs combine information contained in red and NIR bands and normalize external effects, e.g. solar and viewing angles, and internal effects such as soil variation or topographic condition. Some VIs, investigated in this study and described below also included transformed spectral information resulting from expansion of data dimensionality of the original spectral bands.

The normalized difference vegetation index (NDVI) was developed by Tucker [35] and defined by

$$NDVI=\frac{\rho_{nir}-\rho_{red}}{\rho_{nir}+\rho_{red}}$$ (3)

where *ρ*_*nir*_ and *ρ*_*red*_ represents the reflectance of near infrared and red bands respectively. NDVI is a nonlinear index influenced by atmospheric path radiance and affected by an additive noise effect. Since NDVI was derived after image atmospheric correction, it can be used to monitor the seasonal and inter-annual changes in vegetation growth and activity. In particular, it can also help to reduce many forms of multiplicative noise. Huete [36] demonstrated that a potential problem with NDVI is that it is easily affected in the field by background soil. Nevertheless, since this index is widely used for mapping global vegetation cover, the NDVI was included in this study.

In order to cope with the signature biases introduced by soil background, Huete [36] introduced a soil adjusted vegetation index (SAVI) defined as

$$SAVI=\frac{\rho_{nir}-\rho_{red}}{\rho_{nir}+\rho_{red}+L}\left(1+L\right)$$ (4)

where *ρ*_*nir*_ and *ρ*_*red*_ denotes the reflectance of the near infrared and red bands respectively, and “*L*” is an adjustment factor which minimizes the influence of soil brightness in the NDVI. Huete et al. [37] found that the *L* in the SAVI was negatively related to the vegetative percentage in a pixel and suggested that a moderate value of 0.5 for *L* could be applied to general cases for various conditions of green cover. Therefore, the SAVI was included in this study.

Kaufman and Tanré [38] indicated that scattering and absorption caused by aerosols spatially varied in the atmosphere on images where continental, maritime, desert, or heavily vegetation cover varied significantly. To address this issue they developed an atmospherically resistant vegetation index (ARVI) defined by

$$ARVI=\frac{\rho^{*}{}_{nir}-\rho^{*}{}_{rb}}{\rho^{*}{}_{nir}+\rho^{*}{}_{rb}}$$ (5)

where *ρ* ^*^ is a reflectance value with prior correction for molecular scattering and ozone absorption, $\rho_{rb}^{*}=\rho_{red}^{*}-\gamma \left(\rho_{blue}^{*}-\rho_{red}^{*}\right)$ and *a priori* gamma (*γ*) function is introduced into the difference in reflectance between the blue and the red bands ($\rho_{blue}^{*}-\rho_{red}^{*}$) to correct reflectance in the red band ($\rho_{red}^{*}$). The ARVI specified by Eq (5) helps to minimize atmospheric-induced variations in pixel-based vegetation indices as well as stabilize temporal and spatial variations of the indices in atmospheric aerosol content. If the aerosol model is not known *a priori*, the *γ* function is normally set to 1.0 to minimize atmospheric effects.

Gitelson et al. [39] developed the visible atmospherically resistant index (VARI) for remote estimation of vegetation fraction. They found that VARI had minimal sensitivity to atmospheric effects because the estimation error of vegetation fraction was less than 10% in a wide range of atmospheric optical thickness. VARI has a strong linear relationship with vegetation fraction as demonstrated by Viña et al. [40]

$$VARI_{green}=\frac{\rho_{green}-\rho_{red}}{\rho_{green}+\rho_{red}-\rho_{blue}}$$ (6)

where *ρ*_*blue*_, *ρ*_*green*_, and *ρ*_*red*_ represent the reflectance of the blue, green, and red bands respectively.

The enhanced vegetation index (EVI) was also introduced by Huete et al. [41] to optimize the vegetation signal with improved sensitivity in high biomass regions and improved vegetation monitoring through a de-coupling of the canopy background signal and a reduction of atmospheric influences. It is defined by

$$EVI=G\times \frac{\rho^{*}{}_{nir}-\rho^{*}{}_{red}}{\rho^{*}{}_{nir}+C_{1}\cdot \rho^{*}{}_{red}-C_{2}\cdot \rho^{*}{}_{blue}+L}$$ (7)

where *ρ* ^*^ is the atmospherically corrected or partially atmosphere-corrected reflectance, *G* is a gain factor, *L* is the canopy background adjustment factor that addresses nonlinear, differential NIR and red radiant transfer through a canopy, and *C*_1_, *C*_2_ are the coefficients of the aerosol resistance term, which uses the blue band to correct for aerosol influences in the red band. According to Huete et al. [37], the coefficients used in the EVI index are generally set by *L* = 1, *C*_1_ = 6, *C*_2_ = 7.5, and *G* = 2.5.

For vegetation species classification, leaf greenness, percentage variations of mesophyll, and physiological photosynthesis effects need to be considered because these factors might directly or indirectly influence the reflectance of vegetation. According to Larcher [42], the maximum value for net photosynthesis of plants varies dramatically from 17 to 68 (*μ* mol *m* ^−2^ *s* ^−1^). This indicates that trees use the light energy of blue and red spectra differently. Moreover, chlorophyll (the component makes the leaf greenish) is a major factor in photosynthesis. The concentration of leaf pigments generally changes with the seasons and this is the basis of phenological studies. So, it is assumed that signals of blue and green spectra which are usually neglected in forest classification will become essential variables in species recognition. This is the main reason that the three VIs, ARVI, VARI, and EVI were introduced in this study. These spectra indexes have potential in species classification.

### Deriving the spatial texture images from the spectral band images

A spectral image takes into account context, edges, texture and color (tonal variation). The ability to recognize detail is strengthened by considering texture as well as the spatial features of objects of interest. Therefore, each spectral band image produced a spatial texture image which was used in conjunction with the spectral images for data analysis. As a result, an *L*-band remotely sensed image produced an *L*-band spectral-spatial texture (SpecTex) image. These images possess not only spectral information provided by the original spectral band images, but also additional spatial information characterized by texture features in spatial texture images derived from each of *L* spectral band images.

QuickBird image quality is frequently degraded by noise such as random spikes and impulse imperfections [43] which can be limited by using a Lee-sigma filter, a kind of focal filtering analysis [44, 45]. The Lee-sigma filter is a standard deviation-based (sigma) filter that processes data based on statistics calculated within individual filter windows. Unlike a typical low-pass smoothing filter, the Lee-sigma filter produces a texture image which preserves image sharpness and details while suppressing noise. It has been shown to be useful for paddy field extraction from various similar agricultural crops [46] and was therefore appropriate to this study. The value of the pixel being processed, denoted by DN_c_ with the subscript “c” indicating the center pixel in a surrounding window, was replaced by an averaged value calculated from the surrounding pixels in that specific window whose DNs are within a specified threshold. In our experiments, this specific threshold was chosen according to the local noise DN standard deviation, *σ*_*local*_ and the resulting acceptable range of the center pixel DN_c_ was *DN*_*c*_ ± 2*α*_*local*_ [46, 47].

### Assessment of pixel-based classification and accuracy

The supervised maximum likelihood classifier (MLC) [48] was applied to each of the images, i.e, HMS, HMS5VI, SpecTex, and HMSB13, for species classification where the overall kappa coefficient (OKC) and users’ accuracy based species conditional kappa coefficient (SCKC) were calculated based on the error matrix for accuracy assessment. Allouche et al. [49] indicated that the kappa value depended on the prevalence of a particular species. But Vach [50] also demonstrated that of the prevalence of a particular species would have negligible influence. Based on a simulation study of kappa variance, Stehman [51] concluded that the kappa estimator offers little bias even at relatively small sample size. In this study, the true prevalence of a particular species was unknown *a priori*. We applied the stratified random sampling technique to make sure that training and test samples could be selected from each of 40 species. The OKC and SCKC of different datasets were calculated using the same data samples. As a result, if any prevalence dependence existed, its marginal influence would have been the same among those classifications so as to make such classification comparison acceptable.

The introductory section of this paper raised four important issues that can now be addressed. Firstly, the training performance of each of the four classification schemes can be assessed based on the OKC values obtained from using the training samples. The highest OKC value can be considered as the potential highest accuracy (PHA) achievable via high spatial resolution imagery. Secondly, any specific HCA species may be identified if the species conditional kappa coefficient (SCKC) is less than 0.95. Finally, the test accuracy of the classification schemes using MLC classifier is determined from the test samples which are totally independent of the training samples and then classification uncertainty is derived as the difference of training performance and test accuracy. After that, the training performance/test accuracy/classification uncertainty of the four classification schemes were calculated using the same training and test samples and other classifiers such as Mahalanobis distance (MD) [48], spectral angle mapper (SAM) [52], support vector machine (SVM) [53], signature information divergence (SID) [54], and neural net (NN) [48]. A comparison of the classifiers’ efficiency in terms of raising training performance, test accuracy, and uncertainty was then made.

### Statistical Analysis of the classification scheme accuracy

Training performance and test accuracy were based on the OKC value of a classification scheme which combined the input datasets and classifiers. Variations in training performance and test accuracy involved two independent variables (the classifiers and the datasets). In the other words, a particular combination of classifiers and datasets produced performance-OKC and accuracy-OKC was determined via the training and test samples. For a particular type of OKCs, the minimum OKC (OKC_min_) in the classifications using the original HMS image (classification scheme 1) by variant classifiers can be selected as a standard level for statistical comparisons. As a result, the accuracy improvement efficiency (AIE) of any particular combination of classifiers and datasets can be determined as the ratio of OKC increment using Eq (8).

$$\text{AIE}=\left(\text{OKC}-{\text{OKC}}_{\text{min}}\right)/{\text{OKC}}_{\text{min}}$$ (8)

A fixed model-based, 4x6 two-way factorial experiment design defined by Eq (9) was applied to account for the main effects and the interaction of the two variables (classifiers and datasets) on the dependent variable Y, that is the AIE in Eq (8).

$$Y=\beta_{0}+\beta_{1}X_{1}+\beta_{2}X_{2}+\beta_{12}X_{1}X_{2}+\varepsilon$$ (9)

In Eq (9), the coefficients *β*_*i*_ and *β*_*ij*_ represent the effects of the two main variables (X_1_ and X_2_) and the two-variable interactions (X_1_X_2_) on the dependent variable Y, i.e., the AIE, and *ε* is the error term. In the factorial ANOVA analysis, an effect is said to be significant if its coefficient has a probability less than or equal to the significant probability 0.05. Then, a moderate conservative post hoc test, Duncan’s new multiple range test was also applied to interpret multiple comparisons.

## Results

### Performance baseline of multispectral signatures and their derived VI signatures

The overall training performance of the 40-species classification system using dataset 1, the HMS images, was used as a baseline for classification performance. It was evaluated at OKC = 0.58 with a standard deviation 0.20 and the minimal and maximal SCKC values for one of the 40-species were 0.21 and 0.99. If the classification was performed using only dataset 2, i.e., the HMS5VI images, the performance was OKC = 0.48 with a standard deviation 0.21, and SCKC values of every species varied between 0.18 and 0.99. These results demonstrate that baseline classification performance using solely the vegetation indices was around 18% lower than the HMS images. Some of the species could be successfully detected, but the overall results were generally not acceptable if the classification was done using only the original 4-band HMS images or its derived 5-band HMS5VI images.

**Fig 4:**
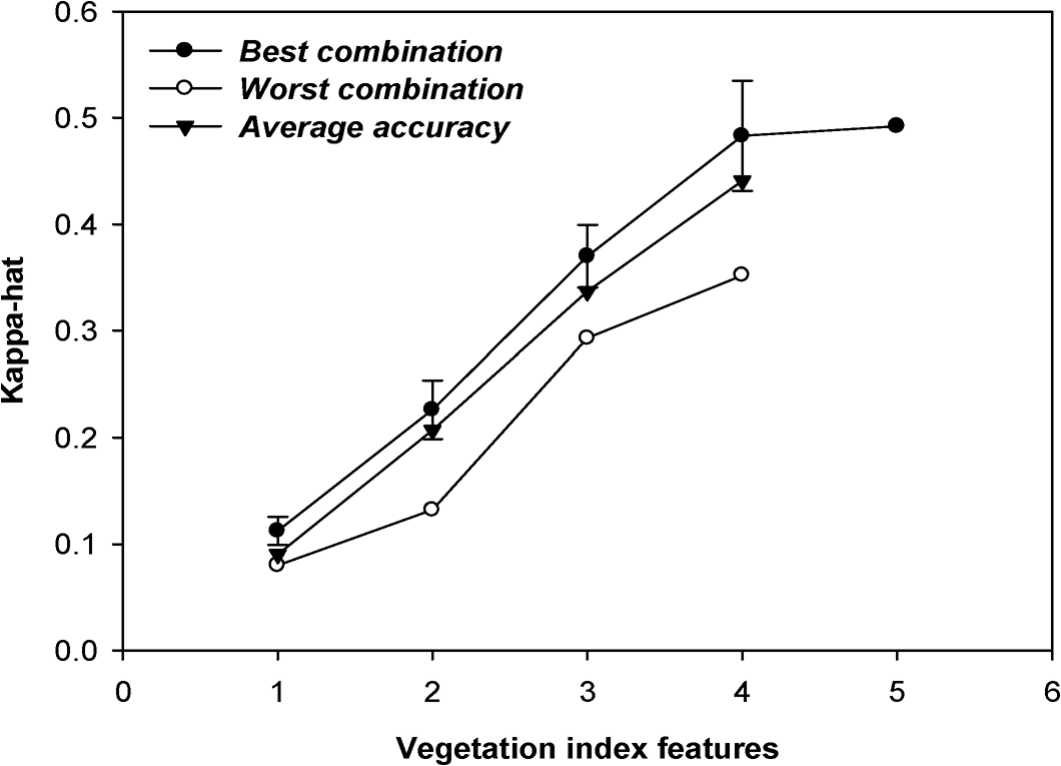
Relationship of classification accuracy using dataset 2 with various numbers of used VI images.

It was also interesting to consider the possibility of a dependency among the VIs in the species classification. Based on our experimental results, the OKC of the tested 40-species classification was below 0.10 when a single VI was used for classification. This is due to similarities between the spectral signatures of these 40 tree species. This situation was somewhat alleviated by including more VIs in the classification, but the improvement in training performance was dependent on the number of VIs used. In order to enhance the findings, various combinations of the five VIs, i.e., NDVI, SAVI, EVI, ARVI, and VARI were used. In particular, classification was performed using combinations of 2-, 3-, 4-, and 5-VIs and then compared and analyzed. Fig 4 shows the OKC of the 40-species classification for dataset 2 using various numbers of used HMS5VI images where the best, average and worst performances were plotted. The improvement in performance appeared to be linear with the number of HMS5VI images used for dataset 2.

### Use of spectral-spatial texture signatures to improve tree species classification

#### Difference between tree-species’ spectral signatures in HMS and SpecTex images

Table 3 lists the transformed divergence (TD) of any two features of the 40 species being compared derived from the 4-band HMS data (the entries in the upper triangle with a gray background) and the 4-band SpecTex data (the entries in the lower triangle with a green background). A blank entry indicates a value of TD = 2000 corresponding to cases where two classes of spectral features are separable. The entry with 1900≤TD<2000 is highlighted in red indicating that good separation was observed between the corresponding two classes. Other cases with TD<1900 meant the two classes of spectral features were not clearly separable. Obviously, the 40 tested tree species whose spectral features are highly similar in the HMS image (most of the entries in the upper triangle of the TD matrix) have values less than 1900. In other words, more than 50% of the species showed a lower level of spectral dissimilarity compared with other species. Fortunately, the poor separation can be significantly improved by using spectral-spatial texture transformation. This can be seen in the lower triangle entries of the TD matrix in Table 3. There are only 12 pairs of between-class TDs among all of the tested 780 pairs that are less than 2000, which was the maximum upper margin of the TD value. Actual TD values between-species are: 1999 for *Z*.*s-S*.*l*, *U*.*p-L*.*f*, *A*.*e-H*.*l*; 1998 for *H*.*c-T*.*o*; 1997 for *S*.*m-B*.*i*; 1996 for *A*.*cu-H*.*l*; 1992 for *S*.*m-T*.*o*; 1985 for *T*.*ch-D*.*r*; 1975 for *F*.*r-C*.*o*; 1961 for *T*.*ch-C*.*o*; 1902 for *R*.*m-M*.*l*; and 1734 for *H*.*c-S*.*m*. According to Jensen [55], Lin [56], and Dwivedi et al. [57], we can conclude that there was only one pair of two-species among all the 40-species whose signatures were inseparable in the SpecTex dataset. The two species are *Haematoxylon campechianum* vs. *Swietenia macrophylla* (TD = 1734).

**Table 3:** Full-bands transformed divergences of between-species in training samples for the original spectral signatures and the spectral-spatial texture signatures. *

	HMS image																			
* *	*A*.*cu*	*T*.*ch*	*P*.*f*	*Z*.*s*	*U*.*p*	*A*.*ca*	*A*.*s*	*R*.*m*	*L*.*S*	*C*.*si*	*C*.*m*	*D*.*s*	*A*.*h*	*M*.*p*	*T*.*ca*	*C*.*g*	*A*.*a*	*H*.*c*	*C*.*f*	*L*.*l*
*A*.*cu*	--	**1983**	**1997**	**1937**	1885		**1955**	**1998**		1769		1798						**1998**	**1912**	1623
*T*.*ch*		--		**1952**	**1986**		**1998**	**1999**	**1998**	1898		**1976**			**1995**			**1993**	**1984**	1783
*P*.*f*			--	**1992**								**1999**								
*Z*.*s*				--	1798		**1968**		**1999**	1827		1745						**1999**	1804	1307
*U*.*p*					--		1887	**1997**	1724	1154	**1997**	1104	**1966**		**1998**	**1966**		1517	702	1059
*A*.*ca*						--			**1955**				1888		**1911**	**1911**		**1980**		
*A*.*s*							--			1411	**1978**	**1911**						**1998**	1772	1803
*R*.*m*								--	**1944**				**1999**					**1998**		**1950**
*L*.*S*									--	**1986**		**1970**	1444	**1964**	**1981**		1804	1365	**1956**	1843
*C*.*si*										--	**1998**	1601	**1999**		**1999**			**1946**	724	827
*C*.*m*											--	**1998**						**1999**	**1999**	
*D*.*s*												--	**1995**		**1999**			1753	1079	1404
*A*.*h*													--	**1998**	1775	**1998**	**1942**	1883	**1992**	**1962**
*M*.*p*														--						
*T*.*ca*															--	**1998**		1785	**1998**	**1999**
*C*.*g*																--		**1991**		**1998**
*A*.*a*																	--	**1999**		
*H*.*c*																		--	1870	1891
*C*.*f*																			--	1124
*L*.*l*																				--
*F*.*r*																				
*S*.*l*				**1999**																
*F*.*e*																				
*S*.*f*																				
*L*.*f*					**1999**															
*A*.*e*																				
*T*.*g*																				
*S*.*m*																		1734		
*S*.*c*																				
*R*.*r*																				
*D*.*r*		**1985**																		
*M*.*l*								**1902**												
*C*.*su*																				
*H*.*l*	**1996**																			
*P*.*r*																				
*K*.*f*																				
*B*.																				
*T*.*o*																		**1998**		
*C*.*o*		**1961**																		
*B*.*i*																				
	**SpecTex image**																			

	HMS image																			
* *	*F*.*r*	*S*.*l*	*F*.*e*	*S*.*f*	*L*.*f*	*A*.*e*	*T*.*g*	*S*.*m*	*S*.*c*	*R*.*r*	*D*.*r*	*M*.*l*	*C*.*su*	*H*.*l*	*P*.*r*	*K*.*f*	*B*.	*T*.*o*	*C*.*o*	*B*.*i*
*A*.*cu*	**1986**	1887		**1970**	**1998**	1833		**1992**		1868	**1991**	**1998**		**1993**						**1988**
*T*.*ch*	**1998**	**1953**	1824	**1993**	**1986**	**1997**		**1975**	**1999**	**1982**	1816			**1962**				**1993**	**1932**	
*P*.*f*				**1993**																
*Z*.*s*	**1999**	**1931**	**1999**	1343	**1992**	1451		**1982**		**1999**	**1975**	**1940**		1859	**1999**					
*U*.*p*	1447	991	**1997**	1738	1642	1661		1593	**1996**	**1965**	1208	**1990**	**1999**	**1968**		**1938**	**1993**	**1990**		1434
*A*.*ca*								**1993**			**1997**					**1999**	**1979**			**1999**
*A*.*s*	**1997**	**1994**		1799	**1997**	**1958**		**1999**		1812	1896			1836						**1987**
*R*.*m*	**1972**	**1999**	**1992**			**1997**		**1956**			**1999**		**1999**					**1999**		**1998**
*L*.*S*	1238	1854	1880		1863	**1976**	**1996**	1341	**1905**		1754		1812			1790	1263	**1914**		1322
*C*.*si*	**1944**	1684	**1989**	1118	1662	1719		**1904**	**1997**	1552	1219			1888	**1999**			**1993**		**1904**
*C*.*m*						**1984**														**1998**
*D*.*s*	**1947**	1533	**1998**	**1909**	**1979**	1780		1661		**1986**	1775	**1932**		**1964**	**1997**	**1967**		**1998**		**1927**
*A*.*h*	**1959**	**1966**	1752		**1978**	**1994**		1418	**1905**		**1902**		1871				1429	1885		**1969**
*M*.*p*																	**1981**			
*T*.*ca*	**1995**	**1965**	1617		**1996**			**1964**	**1992**		**1975**		**1999**				**1942**	**1949**	**1984**	**1999**
*C*.*g*	**1997**					**1999**		**1919**			**1967**					**1998**				**1984**
*A*.*a*	**1999**		**1976**						**1950**				1869				1408	1987		1998
*H*.*c*	1429	1001	**1901**	**1997**	**1944**	**1925**		1419	**1790**		1704		**1945**			**1993**	1849	1847		1135
*C*.*f*	**1958**	1603	**1992**	1539	1653	1872		1727	**1999**	**1938**	1069	**1994**		**1922**	**1999**	1818	**1999**	**1993**		**1917**
*L*.*l*	1655	1269	**1957**	1610	1572	1446		1623	**1982**	1885	1416	**1998**	**1999**	1859			**1995**	**1971**		1808
*F*.*r*	--	1632	**1982**	**1994**	1895	1869		1724	**1953**	**1996**	1765		**1980**			**1998**	**1964**	**1979**		1001
*S*.*l*		--	**1988**	1872	1705	1543		1722	**1980**	**1980**	1515	**1998**		1997		**1999**	**1994**	**1955**		1814
*F*.*e*			--		**1990**	**1999**		**1941**	1684		**1931**		1167				1721	955	**1998**	**1988**
*S*.*f*				--	**1920**	1566				**1981**	**1916**			1823						**1997**
*L*.*f*					--	**1938**		**1946**	**1900**	**1992**	1124		**1998**	**1998**		**1998**	**1984**	**1987**		**1955**
*A*.*e*						--		**1958**		**1990**	1948			**1938**				**1999**		**1929**
*T*.*g*							--				**1999**						**1999**			
*S*.*m*								--	**1971**		1567		**1969**			**1912**	1799	1883		1628
*S*.*c*									--		**1936**		1729				1756	1477		**1924**
*R*.*r*										--	**1967**			**1998**						**1996**
*D*.*r*											--		**1997**	**1981**		1897	**1953**	**1967**		1702
*M*.*l*												--								
*C*.*su*													--				1471	1250		**1981**
*H*.*l*						**1999**								--						
*P*.*r*															--					
*K*.*f*																--	**1992**			**1987**
*B*.																	--	1440		**1921**
*T*.*o*								**1992**										--		**1969**
*C*.*o*	**1975**																		--	
*B*.*i*								**1997**												--
	**SpecTex image**																			

*: the “blank” entries have TD = 2000, the bold number highlighted 1900<TD≤2000 represents spectral features are good separable.

#### Improvement of training performance after integrating HMS images, VIs, and spectral-spatial texture images

The QuickBird HMS data pixel size is smaller than one meter, so an overstorey tree crown will encompass more than one pixel of contiguous space. The height variations and the vertical structure changes in crown pixels might introduce significant noise which may make spectral signatures of tree species complicated. Applying the Lee-sigma filter to the original image can remove the noise and produce a more homogeneous spectral-spatial texture image for improved recognition of species spectra. The spectral-spatial texture not only improved the statistical distance between species, but also maximized their signature divergences. These benefits are demonstrated in Table 3. In addition, Table 4 shows the results of a comprehensive comparative study among the multiple species using four data sets; (1) dataset 1: four original bands, HMS (2) dataset 2: five VI images, HMS5VI (3) dataset 3: four spectral-spatial textural images, SpecTex and (4) dataset 4: 13 layer of images (HMS13B) which are combined by the HMS, HMS5VI, and SpecTex images.

**Table 4:** Comparisons of the training-samples-based SCKC for the five HCA-species and the OKC for all 40 species in the classification using various data sets.

Data sets	SpecTex4 bands	SpecTex+ARVI	SpecTex+EVI	SpecTex+NDVI	SpecTex+SAVI	SpecTex+VARI	HMS13B13bands
SCKC							
*C.o*	0.68	0.77	0.80	0.76	0.79	0.94	1.00
*H.l*	0.84	0.84	0.93	0.86	0.93	1.00	1.00
*P.f*	0.94	0.94	0.96	0.94	0.96	0.94	1.00
*R.m*	0.90	0.92	0.94	0.90	0.91	1.00	1.00
*S.m*	0.94	0.95	0.98	0.95	0.98	0.97	1.00
OKC							
40 species	0.9861	0.9898	0.9931	0.9900	0.9925	0.9945	0.9971

S1 Table, shows the SCKC of the 40 tree species. When the HMS image was used for classification, only the species *T*.*ca* had a nearly perfect conditional accuracy with SCKC = 0.99 compared to the species *P*.*f* with SCKC = 0.94, three species: *B*., *M*.*l*, and *P*.*r* with 0.80<SCKC<0.90, 7-species: *A*.*s*, *B*.*i*, *D*.*r*, *L*.*l*, *R*.*m*, *S*.*c*, and *T*.*o* with 0.70<SCKC<0.80, 5 species: *D*.*s*, *F*.*e*, *H*.*c*, *M*.*p*, and *U*.*p* with 0.70<SCKC<0.60; and the others with SCKC less than 0.60. It was also observed that the misclassification rate was increased when the classification was made on the 5-band VI image. Two of the tested species, i.e, *B*. and *T*.*ca* had good accuracy (SCKC>0.90) and 9 species: *D*.*r*, *F*.*r*, *M*.*l*, *P*.*r*, *R*.*m*, *R*.*r*, *S*.*c*, *S*.*l*, and *T*.*o* were classified with a relatively better accuracy (SCKC>0.6), most of the 40 species were classified at very low SCKC values compared to the results produced by using the 4-band original image.

The SpecTex image signatures of the 40 tree species were nearly correctly recognized (SCKC≥0.95) with the exception of 5 species (Table 4) whose SCKC values ranged from 0.68–0.94. Those five species are *C*.*o*: *Cananga odorata*, *H*.*l*: *Heritiera littoralis*, *R*.*m*: *Ravenala madagascariensis*, *S*.*m*: *Swietenia macrophylla*, *and P*.*f*: *Palaquium formosanum*; they can be considered as the hard to classify accurately (HCA) species. Fortunately, those HCA species can be successfully recognized using the HMS13B: the integrated image of HMS, HMS5VI, and SpecTex.

Combining the 5-VI (NDVI, SAVI, ARVI, VARI, and EVI) into one image (HMS5VI) only achieved a 0.48 OKC valuewhich was worse than the 0.58 OKC obtained using the original 4-band HMS image. The reduced OKC of 0.10 was probably due to the fact that each of the VIs use only a partial set of spectral signatures from the HMS image. On the other hand, using only 4-band SpecTex image achieved as high as OKC = 0.98 with improvements of 104% and 69% compared to the 5-band HMS5VI image and the original 4-band HMS image respectively. By contrast, all the tested 40 tree species were successfully classified with an overall performance OKC = 0.98 when the 4-band SpecTex image was used for classification. Moreover, an overall performance OKC = 0.9971≈1.00 was achieved when the integrated image HMS13B was used for classification (Table 4).

## Discussion

### VI as the critical signature for recognizing HCA species

The two issues that were of particular interest in this study were mentioned previously, (1) What is the potential highest accuracy (PHA) that can be achieved using the high spatial resolution satellite images? (2) What is the critical marginal signature that could be used to improve species classification up to the PHA level? These signatures can be used as a guide for figuring out key features that contain crucial information for recognition of those species that are difficult to discriminate, as well as features that contain redundant information which can be removed from the classification. To satisfy such needs, we have to understand the potential of the various combinations of high spatial resolution images.

Overall performance was OKC = 0.99 for the 40 species. Very few pixels were misclassified in terms of between-species discrimination (TD can be seen in the lower triangle matrix in Table 3). PHA for each of the tested species is shown in column “HMS13B” next to “SpecTex” in S1 Table. Five species proved hard to classify accurately (HCA). They were, *Cananga odorata*, *Heritiera littoralis*, *Palaquium formosanum*, *Ravenala madagascariensis*; and *Swietenia macrophylla* and are highlighted in S1 Table. Two data sets, those derived from the SpecTex images and those derived from the HMS13B images proved to be effective according to the results in S1 Table. In addition to these two data sets, data sets that combined SpecTex images with one VI image were included to see whether or not including a VI image into the 4 spectral-spatial texture images could improve classification performance. VARI was shown to be a very good predictor for vegetation fraction estimation in [39] and [40]. The VARI vegetation index can organize blue, green, and red spectra more efficiently to present leaf chlorophyll, color information and crown coverage. Accordingly, it is suggested that VARI is able to retain heterogeneous spectra of those visible spectral bands and thus contribute better marginal performance for species classification as shown in Table 4. Adding the VARI to the SpecTex images significantly improved the training performance, especially in three species, *Cananga odorata*, *Heritiera littoralis*, and *Ravenala madagascariensis*; while another four species showed little additional performance. Thus, classification using HMS13B images, which integrated heterogeneous signatures from HMS, HMS5VI, and SpecTex, offered the highest SCKC and OKC performance for the tree species tested in this study (Table 4).

### Species classification uncertainty

**Fig 5:**
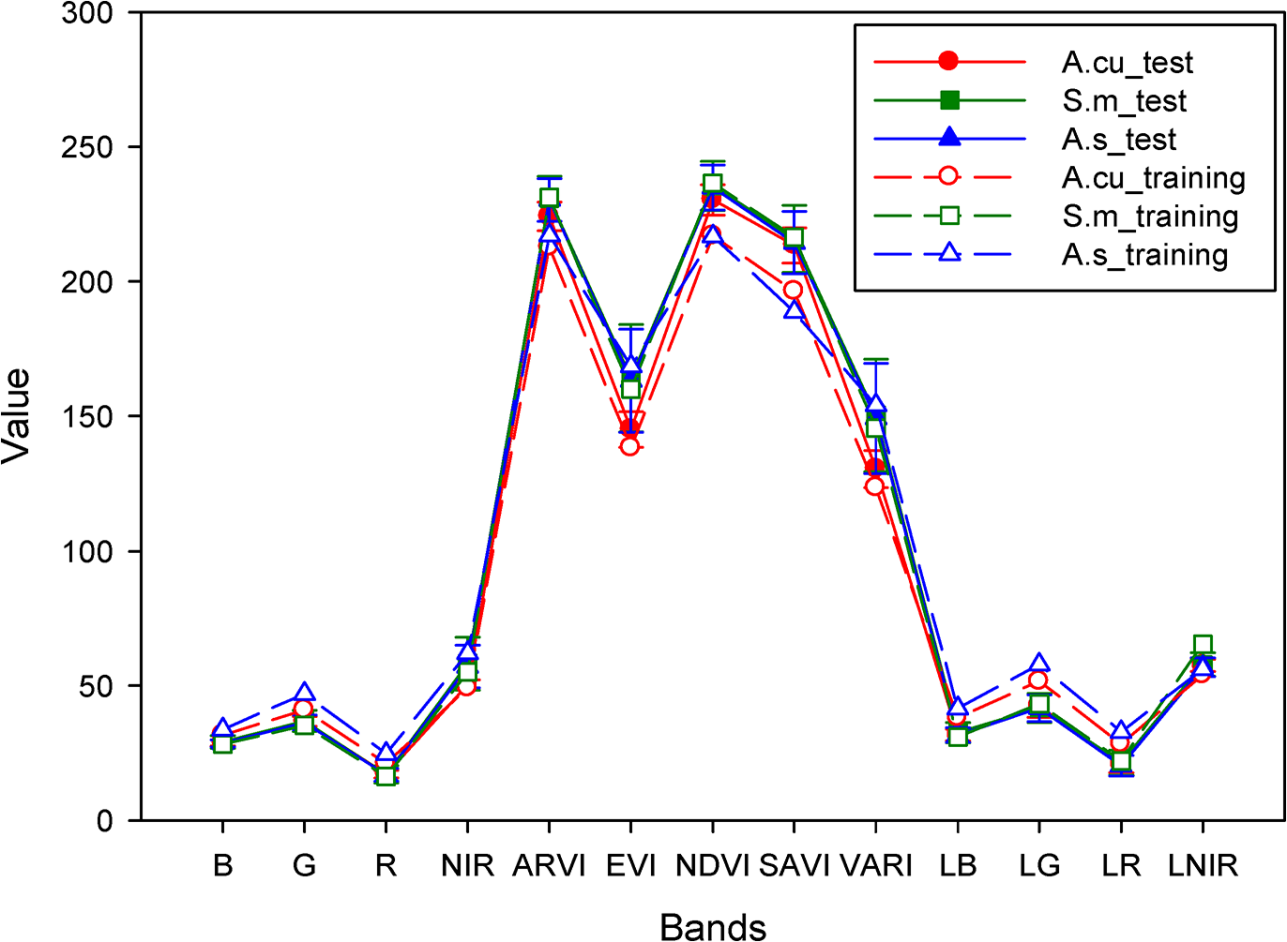
An example of the species classification uncertainty caused by spectral signatures variations.

Assessment of the training samples in the previous section showed good potential for species classification using HMS13B images. However, a large uncertainty of species mapping was still visible in the integrated spectral-spatial texture QuickBird images because of unsatisfactory overall kappa accuracy (OKC<0.20) obtained in the test samples of those species. Based on ground checks after classification, examples of individual trees of the same species were observed with different foliage color and foliage density in their test and training samples. The amount of leaf greenness reflects the chlorophyll concentration and the ability of leaf photosynthesis [58]. Foliage density meanwhile is the most widely used and most rapid method of determining forest health [59]. High variations of reflectance caused by inter- and intra-canopy gaps and leaf color changes were the major factors that reduced species mapping accuracy. Fig 5 demonstrates the signature variations of tree species in the study site. In this figure, signatures of the training samples and test samples of *Araucaria cunninghamii*, *Alstonia scholaris*, and *Swietenia macrophylla* significantly overlap each other.

**Fig 6:**
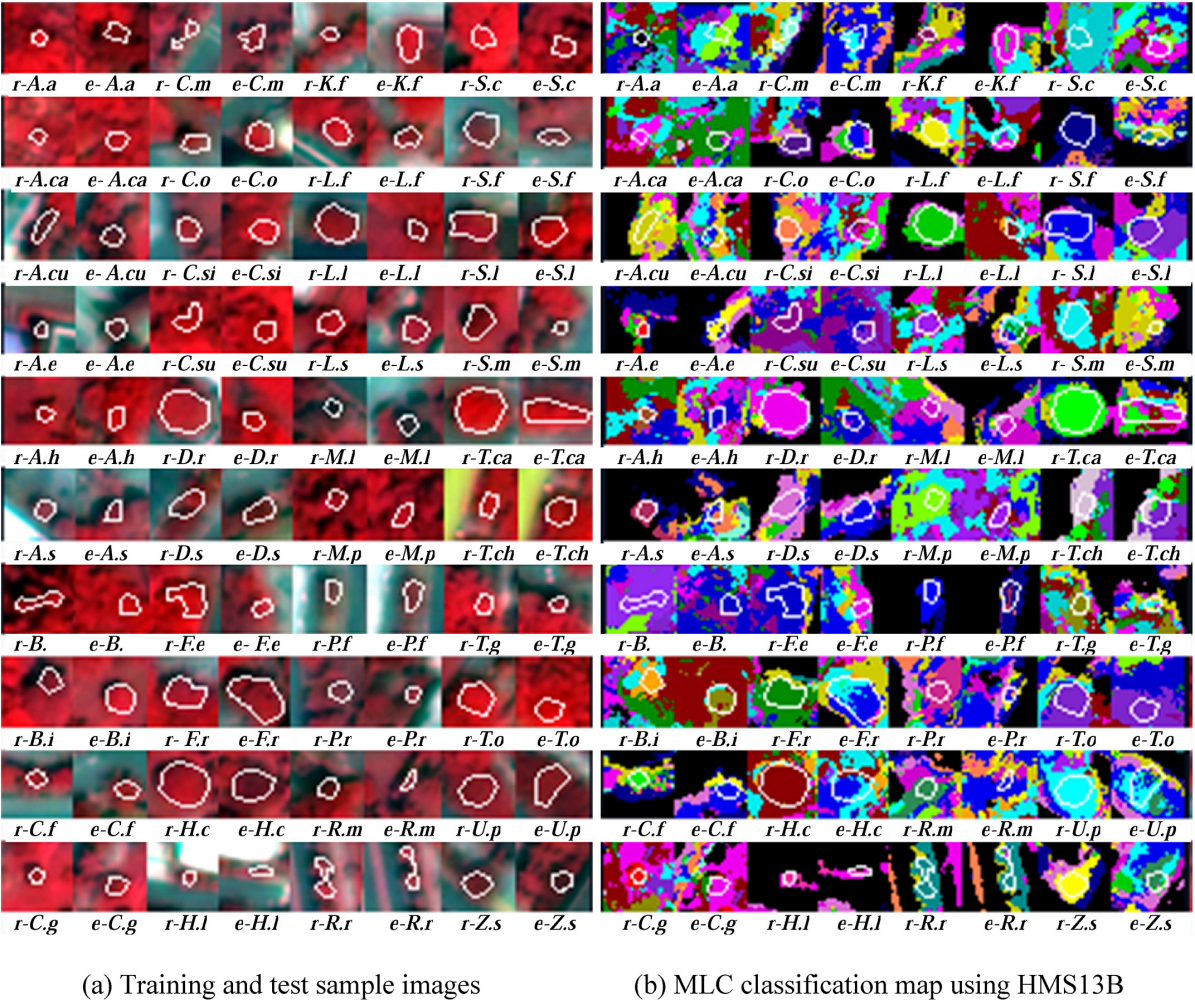
A magnified view of the images (a) and classifications result of tree species (b). Below each image, the prefixes “r-” and “e-” before the code of tree species denote training and test samples respectively. The white polygon in an image depicts the training samples or test samples of that specific species.

The uncertainty of tree species classification can also be examined visually by a close look at Fig 6. It can be seen, that the training samples of the 40 species highlighted by polygons (denoted r-Species code) were mostly classified accurately; however, the test samples (abbreviated e-Species code) were mostly confused. Most of the test samples of the 40 species showed a diverse colors indicating the test accuracy was not optimistic and the classification of a large number of tree species suffered from high uncertainty that was probably caused by tree physiology and physical properties of tree crowns. In contrast to Fig 6B, S2 Table shows the test-samples-based SCKC of the MLC classification. It reveals a serious problem of misclassification in the species classification. Most of the 40 species have a SCKC less than 0.20 in every classification scheme. In other words, the number of species with SCKC≥0.20 was 6, 5, 5, and 9 for classification schemes 1, 2, 3, and 4 respectively. The largest SCKC was in classification scheme 4 and equaled 1.00 which was greater than 0.73, 0.39, and 0.53, the largest SCKC in classification scheme 1, 2, and 3. A difference between SCKC values in training- and test-samples has also been demonstrated.

### Comparison of classifiers and input datasets on classification efficiency

**Fig 7:**
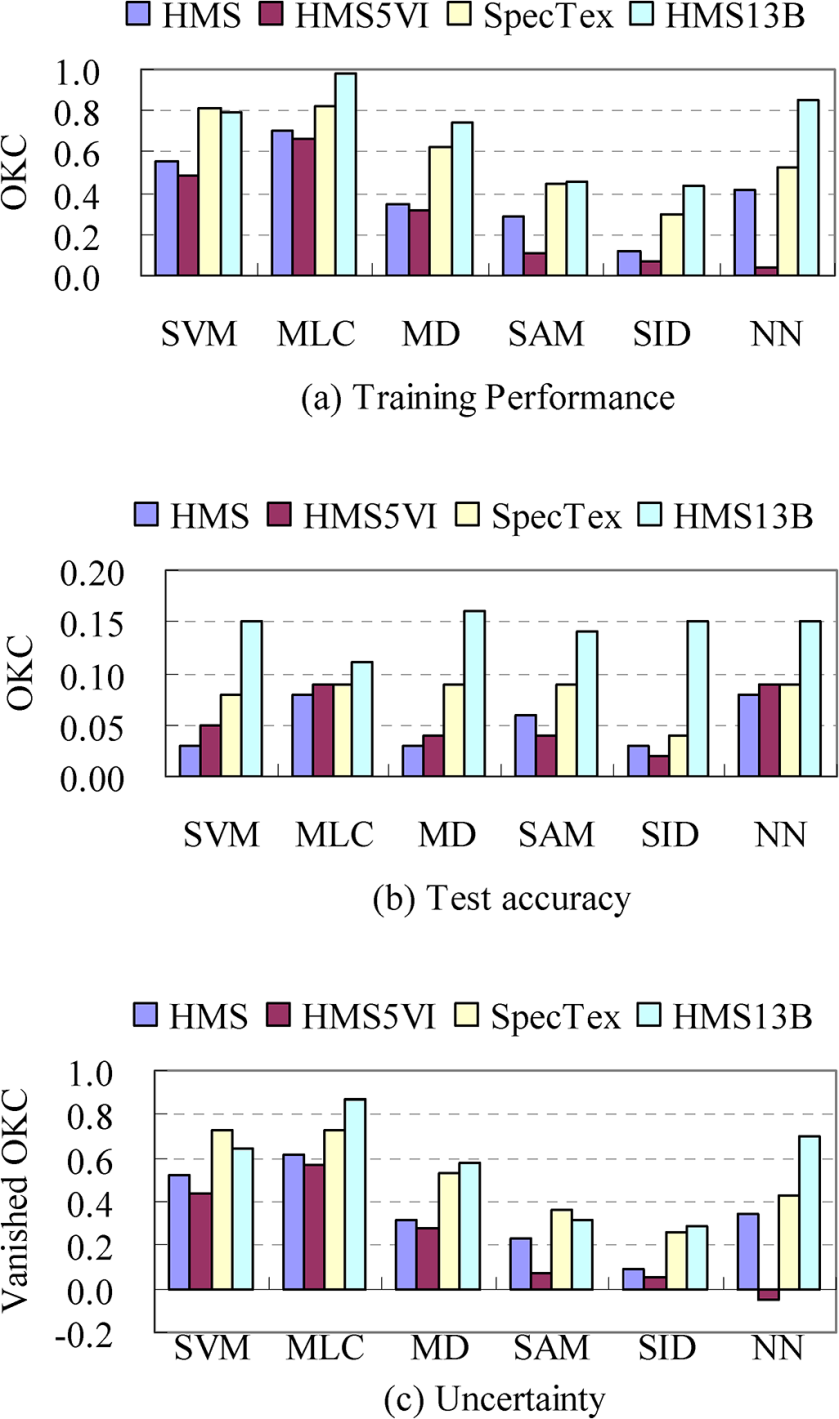
A comparison of the training performance, test accuracy, and uncertainty among classifiers in variant classification schemes.

Fig 7A–7C shows the diversity of training performance, test accuracy, and classification uncertainty in the classifiers. This kind of variation also appeared in the four datasets by variant classifiers. The highest and the lowest training performance (0.98 and 0.04) were achieved by MLC and NN that was carried out using the HMS13B and HMS5VI images respectively. The average and standard deviation of the classifiers’ performance derived from the four datasets are MLC 0.79±0.16, SVM 0.66±0.16, MD 0.51±0.21, NN 0.46±0.33, SAM 0.33±0.16, and SID 0.23±0.17. Differences between performance and accuracy were repeated in each of the other classifiers. The classifiers’ test accuracy was NN 0.10±0.03, MLC 0.09±0.01, MD 0.08±0.06, SVM 0.08±0.05, SAM 0.08±0.04, and SID 0.06±0.06. Classification uncertainty was defined as the loss of OKC which was calculated as the difference between training performance and test accuracy. The vanished OKC of the classifiers was MLC 0.70±0.13, SVM 0.58±0.13, MD 0.43±0.15, NN 0.36±0.31, SAM 0.25±0.13, and SID 0.17±0.12.

Based on the minimum OKC of classification using the HMS image (classification scheme 1), that is performance-OKC = 0.12 and accuracy-OKC = 0.03 for the classifier SID, a further statistical test of the OKC increment achieved by all combination of classifiers and datasets was carried out by a two-way ANOVA test. As shown in S3 Table, the effect of classifiers (F = 3.234, sig. probability = 0.023) and datasets (F = 10.320, sig. probability<0.001) on the change of AIE was significant, and the interaction was insignificant (F = 0.215, sig. probability = 0.998). In addition, S4 and S5 Tables showed the Duncan’s grouping of AIEs in the aspect of classifiers and datasets respectively. As shown in S4 Table, the classifications using the HMS13B image (classification scheme 4) achieved the highest value of AIE = 4.35 which is significantly greater than the others. AIE of the SpecTex (classification scheme 3) was 2.78 which is statistically equal to 1.55 the AIE of the HMS (classification scheme 1) at the significant probability = 0.067. Of all the datasets, the HMS5VI has the smallest AIE of 1.09 (S4 Table). S5 Table shows the AIE of the classifiers ranging from 0.97 to 3.83. Although the values have been graded into 3 groups by Duncan’s test, the AIE values were mostly insignificant in relation to each other. But the MLC and SVM, with AIE 3.83 and 3.04, respectively, were significantly higher than the SID. In brief, the AIE statistical test suggested the HMS13B image when combined with the MLC or SVM classifier was able to achieve improved multiple species classification results.

### Recommendations for further study

The classifiers MLC, SVM, SAM, MD, SID, and NN have been intensively applied in LULC and forest classifications. In this study, excellent training performance was obtained by integrating the high spatial resolution multispectral image, and their derived vegetation indices and texture images. Unfortunately, no improvement in accuracy could be achieved using the test samples. This indicates that high variation of species spectra and wide-spread forest are major factors in performance. In addition, a possible variation of phenological events and/or tree health might cause changes in individual trees of a particular species leading the species classification into a more complicated situation. In these cases, additional spectral signatures and nonparametric methods might be helpful. This may be worthy of further study in the future.

In tropical and sub-tropical forest, many species compete for dominance in the overstorey canopy. Changes in the composition of the overstorey canopy might influence the extent of biodiversity, and the structure of the forest community, and succession. As Chytrý et al. [60] indicated, vegetation surveys that define vegetation types and help understand differences among them are essential for basic ecological research and applications in biodiversity conservation and environmental monitoring. To improve our understanding of forest data, particularly, tree species composition, efforts in using remote sensing imagery to effectively expand more heterogeneous spectra and textural information could be explored further. Imagery with high spatial resolution and hyperspectral signatures in the visible-short wave infrared region would also be highly desirable.

WorldView-2 is capable of collecting bands of Coastal, Blue, Green, Yellow, Red, Red Edge, Near-IR1, and Near-IR2. WorldView-3 is able to collect an additional 8 SWIR (Short-Wave Infrared) bands and 12 CAVIS (Clouds, Aerosols, Vapors, Ice, and Snow) bands. These two HSR multispectral satellites offer more diverse spectral information than Quickbird-2 multispectral image. Therefore, a variety of additional vegetation indices could be derived to explore extra spectral information to diagnose or describe properties of tree species. For example, a new spectral feature (effective chlorophyll index, ECI) developed by Lin et al. [58] is an index that derived by blue, red, and red edge spectra was approved to be able to accurately predict total chlorophyll content of foliage and therefore can help to identify the variations of greenness in tree crown or canopy. In other words, by taking of the availability of diverse spectral bands at variable wavelengths highly accurate mapping of tree species using satellite/airborne remote sensing images is achievable.

Although, object-based classifiers have been successfully applied in LULC and forest mapping recently [54, 61, 62], there still a big challenge to overcome the high complexity of tree-species spectra to reduce classification uncertainty or accomplish a classification results with great agreement of both training performance and test accuracy. Tree spectral reflectance varies in respect to factors such as crown structure, phenology [61], and physiological and disease stress [63] which vary with time as well. If an object-based methodology can integrates spectral information with crown-shape parameters, it will greatly assist the process of tree mapping and more accurately fit the needs of forest ecosystem management. This may provide a fruitful direction for future studies.

## Conclusions

There has been a lot of interest recently in species classification using remote sensing techniques, but few studies have attempted to classify a large number of tree species using satellite images. This paper introduced a novel remote-sensing-based method for the classification of 40 tree species in subtropical forest using high-spatial-resolution QuickBird multispectral images. Classification performance and accuracy was measured using OKC and SCKC. Several interesting findings emerged from the study and some potential issues of concern were raised. Suggestions are offered below:

- The atmospherically corrected high-spatial-resolution QuickBird multispectral image is not able to identify 40 tree species simultaneously. Although the vegetation indices NDVI, SAVI, EVI, ARVI, and VARI help to supplement the regular multiple spectra with additional information, without the original spectral signatures, no improvement could be gained in classification.
- Texture transformation is also capable of retrieving additional spectral information in respect to the spectra of a multispectral image. Using a dimensionality expansion technique to integrate multiple images is helpful to gather diverse spectral information and for deriving tree species signatures. However, this classification scheme still has problems with tree classification outside the training samples and can not achieve a general level of accuracy, for example an overall kappa coefficient of 0.80 or better in land use and land cover (LULC) classification.
- Ground survey observations revealed some substantial differences among individual samples of the same tree species such as the leaf density of tree crown, leaf color, and the surrounding trees. Leaf color and the density of the tree crown are major factors regulating tree physiology and may influence the spectral absorption and reflection features. In addition, low foliage density creates gaps in the tree crown or canopy and also increases the amount of light reaching the ground. The effect increases the influence of the background material. As a result, there is a much greater variation in spectral reflectance that may contribute to misclassification. This issue presents a significant challenge to forest species mapping using remote sensing which we intend to investigate further in future studies.
- Integrating high-spatial-resolution canopy height model data and satellite images with diverse spectral information could further enrich the spectral signatures and spatial texture data specific to individual tree species and therefore obtain a higher potential accuracy rate in overstorey-canopy species mapping.

## Supporting Information

S1 Table The training-sample-based species conditional kappa coefficient (SCKC) of each species for the MLC classification using variant data sets^#)^. (DOC)

S2 Table The test-sample-based species conditional kappa coefficient (SCKC) of each species for the MLC classification using variant data sets. (DOC)

S3 Table ANOVA test of the accuracy improvement efficiency (AIE) of tree species classification. (DOC)

S4 Table Duncan’s new multiple range method determined grouping for the average AIE of the dataset used in tree species classification. (DOC)

S5 Table Duncan’s new multiple range method determined grouping for the average AIE of the classifiers in tree species classification. (DOC)
