# Supplementary material for: Classification of Tree Species in Overstorey Canopy of Subtropical Forest Using QuickBird Images
Source: PLoS One. 2015 May 15;10(5):e0125554. doi: 10.1371/journal.pone.0125554 (PMC4433356; doi:10.1371/journal.pone.0125554)
Supplement: S1 Table — (DOC) [file pone.0125554.s001.doc]

**Table S1.** The training-sample-based species conditional kappa coefficient (SCKC) of each species for the MLC classification using variant data sets^#)^.

| Species  codes | HMS  4 bands | HMS5VI  5 bands | SpecTex  4 bands | HMS13B  13 bands | Species  codes | HMS  4 bands | HMS5VI  5 bands | SpecTex  4 bands | HMS13B  13 bands |
| --- | --- | --- | --- | --- | --- | --- | --- | --- | --- |
| *A.a* | 0.27 | 0.20 | 1.00 | 1.00 | *K.f* | 0.39 | 0.20 | 1.00 | 1.00 |
| *A.ca* | 0.38 | 0.47 | 1.00 | 1.00 | *L.f* | 0.32 | 0.29 | 1.00 | 1.00 |
| *A.cu* | 0.52 | 0.32 | 1.00 | 1.00 | *L.l* | 0.71 | 0.59 | 1.00 | 1.00 |
| *A.e* | 0.45 | 0.25 | 1.00 | 1.00 | *L.s* | 0.28 | 0.24 | 0.99 | 0.99 |
| *A.h* | 0.21 | 0.27 | 1.00 | 1.00 | *M.l* | 0.82 | 0.65 | 1.00 | 1.00 |
| *A.s* | 0.75 | 0.58 | 0.96 | 1.00 | *M.p* | 0.66 | 0.35 | 1.00 | 1.00 |
| *B.* | 0.89 | 0.91 | 1.00 | 1.00 | *P.f* | 0.94 | 0.58 | 0.94 | 1.00 |
| *B.i* | 0.72 | 0.63 | 1.00 | 0.99 | *P.r* | 0.84 | 0.74 | 1.00 | 1.00 |
| *C.f* | 0.22 | 0.18 | 1.00 | 1.00 | *R.m* | 0.71 | 0.71 | 0.90 | 1.00 |
| *C.g* | 0.34 | 0.28 | 1.00 | 1.00 | *R.r* | 0.56 | 0.75 | 1.00 | 1.00 |
| *C.m* | 0.59 | 0.52 | 1.00 | 1.00 | *S.c* | 0.74 | 0.68 | 1.00 | 1.00 |
| *C.o* | 0.55 | 0.35 | 0.68 | 1.00 | *S.f* | 0.59 | 0.49 | 1.00 | 1.00 |
| *C.si* | 0.28 | 0.20 | 1.00 | 1.00 | *S.l* | 0.50 | 0.66 | 0.99 | 0.99 |
| *C.su* | 0.53 | 0.42 | 0.99 | 1.00 | *S.m* | 0.48 | 0.52 | 0.94 | 1.00 |
| *D.r* | 0.79 | 0.75 | 1.00 | 1.00 | *T.ca* | 0.99 | 0.99 | 0.97 | 0.98 |
| *D.s* | 0.60 | 0.53 | 1.00 | 1.00 | *T.ch* | 0.54 | 0.38 | 1.00 | 1.00 |
| *F.e* | 0.65 | 0.58 | 1.00 | 1.00 | *T.g* | 0.48 | 0.27 | 1.00 | 1.00 |
| *F.r* | 0.48 | 0.62 | 0.99 | 1.00 | *T.o* | 0.79 | 0.65 | 1.00 | 1.00 |
| *H.c* | 0.62 | 0.48 | 0.99 | 0.99 | *U.p* | 0.60 | 0.58 | 0.98 | 1.00 |
| *H.l* | 0.31 | 0.18 | 0.84 | 1.00 | *Z.s* | 0.57 | 0.45 | 0.99 | 1.00 |

^#^: The highlighted records indicate the HAC species.
