# Supplementary material for: Classification of Tree Species in Overstorey Canopy of Subtropical Forest Using QuickBird Images
Source: PLoS One. 2015 May 15;10(5):e0125554. doi: 10.1371/journal.pone.0125554 (PMC4433356; doi:10.1371/journal.pone.0125554)
Supplement: S2 Table — (DOC) [file pone.0125554.s002.doc]

**Table S2.** The test-sample-based species conditional kappa coefficient (SCKC) of each species for the MLC classification using variant data sets.

| Species  codes | HMS  4 bands | HMS5VI  5 bands | SpecTex  4 bands | HMS13B  13 bands | Species  codes | HMS  4 bands | HMS5VI  5 bands | SpecTex  4 bands | HMS13B  13 bands |
| --- | --- | --- | --- | --- | --- | --- | --- | --- | --- |
| *A.a* | 0.00 | 0.00 | 0.00 | 0.00 | *K.f* | 0.00 | 0.01 | 0.01 | 0.00 |
| *A.ca* | 0.10 | 0.02 | 0.04 | 0.00 | *L.f* | 0.00 | 0.00 | 0.00 | 0.00 |
| *A.cu* | 0.68 | 0.20 | 0.00 | 0.32 | *L.l* | 0.13 | 0.05 | 0.00 | 0.00 |
| *A.e* | 0.42 | 0.14 | 0.00 | 0.00 | *L.s* | 0.00 | 0.00 | 0.00 | 0.00 |
| *A.h* | 0.00 | 0.02 | 0.04 | 0.00 | *M.l* | 0.34 | 0.15 | 0.00 | 0.63 |
| *A.s* | 0.12 | 0.00 | 0.00 | 0.00 | *M.p* | 0.03 | 0.00 | 0.23 | 0.00 |
| *B.* | 0.00 | 0.00 | 0.00 | 0.00 | *P.f* | 0.00 | 0.00 | 0.05 | 0.00 |
| *B.i* | 0.00 | 0.01 | 0.00 | 0.00 | *P.r* | 0.05 | 0.04 | 0.00 | 0.00 |
| *C.f* | 0.00 | 0.03 | 0.01 | 0.00 | *R.m* | 0.00 | 0.00 | 0.00 | 0.00 |
| *C.g* | 0.05 | 0.00 | 0.00 | 0.00 | *R.r* | 0.26 | 0.03 | 0.16 | 0.71 |
| *C.m* | 0.06 | 0.04 | 0.00 | 0.00 | *S.c* | 0.11 | 0.00 | 0.00 | 0.00 |
| *C.o* | 0.07 | 0.04 | 0.09 | 0.10 | *S.f* | 0.00 | 0.04 | 0.01 | 0.21 |
| *C.si* | 0.03 | 0.01 | 0.00 | 0.00 | *S.l* | 0.00 | 0.00 | 0.00 | 0.07 |
| *C.su* | 0.73 | 0.34 | 0.41 | 0.15 | *S.m* | 0.00 | 0.00 | 0.00 | 0.00 |
| *D.r* | 0.00 | 0.00 | 0.00 | 0.14 | *T.ca* | 0.12 | 0.23 | 0.00 | 0.29 |
| *D.s* | 0.02 | 0.17 | 0.15 | 0.14 | *T.ch* | 0.50 | 0.27 | 0.53 | 0.30 |
| *F.e* | 0.00 | 0.00 | 0.00 | 0.00 | *T.g* | 0.08 | 0.00 | 0.00 | 0.00 |
| *F.r* | 0.02 | 0.12 | 0.00 | 0.00 | *T.o* | 0.00 | 0.01 | 0.04 | 1.00 |
| *H.c* | 0.09 | 0.17 | 0.22 | 0.12 | *U.p* | 0.29 | 0.39 | 0.35 | 0.57 |
| *H.l* | 0.00 | 0.00 | 0.06 | 0.31 | *Z.s* | 0.00 | 0.00 | 0.00 | 0.00 |
