# Supplementary material for: Classification of Tree Species in Overstorey Canopy of Subtropical Forest Using QuickBird Images
Source: PLoS One. 2015 May 15;10(5):e0125554. doi: 10.1371/journal.pone.0125554 (PMC4433356; doi:10.1371/journal.pone.0125554)
Supplement: S3 Table — (DOC) [file pone.0125554.s003.doc]

**Table S3.** ANOVA test of the accuracy improvement efficiency (AIE) of tree species classification.

| Sources | Sum of square | df | Mean square | F | Sig. probability |
| --- | --- | --- | --- | --- | --- |
| Corrected model^#^ | 124.311 | 23 | 5.405 | 2.189 | 0.031 |
| Intercept | 286.017 | 1 | 286.017 | 115.865 | <0.001 |
| Datasets | 76.423 | 3 | 25.474 | 10.320 | <0.001 |
| Classifiers | 39.911 | 5 | 7.982 | 3.234 | 0.023 |
| Datasets * Classifiers | 7.978 | 15 | .532 | .215 | 0.998 |
| Error | 59.245 | 24 | 2.469 |  |  |
| Total | 469.573 | 48 |  |  |  |
| Corrected total | 183.556 | 47 |  |  |  |
| #: R-square = 0.677 | | | | | |
