# Supplementary material for: Classification of Tree Species in Overstorey Canopy of Subtropical Forest Using QuickBird Images
Source: PLoS One. 2015 May 15;10(5):e0125554. doi: 10.1371/journal.pone.0125554 (PMC4433356; doi:10.1371/journal.pone.0125554)
Supplement: S4 Table — (DOC) [file pone.0125554.s004.doc]

**Table S4.** Duncan’s new multiple range method determined grouping for the average AIE of the datasets used in tree species classification.

| Datasets | HMS5VI | HMS | SpecTex | HMS13B |
| --- | --- | --- | --- | --- |
| AIE | 1.09 | 1.55 | 2.78 | 4.35 |
| Grouping^#^ | a | a, b | b | c |

^#^: Alphabetical codes in the entries of “Grouping” stand for the grouping of the mean value of AIE as determined by Duncan’s test. Accuracy Increment values with the same letter indicating that there is no difference between them at the 0.05 probability level.
