# Supplementary material for: Classification of Tree Species in Overstorey Canopy of Subtropical Forest Using QuickBird Images
Source: PLoS One. 2015 May 15;10(5):e0125554. doi: 10.1371/journal.pone.0125554 (PMC4433356; doi:10.1371/journal.pone.0125554)
Supplement: S5 Table — (DOC) [file pone.0125554.s005.doc]

**Table S5.** Duncan’s new multiple range method determined grouping for the average AIE of the classifiers in tree species classification.

| Classifiers | SID | SAM | MD | NN | SVM | MLC |
| --- | --- | --- | --- | --- | --- | --- |
| AIE | 0.97 | 1.74 | 2.45 | 2.61 | 3.04 | 3.83 |
| Grouping^#^ | a | a, b, | a, b, c | a, b, c | b, c | c |

^#^: same as Table S3.
